# Data showing the compositional complexity of the mitochondrial proteome of a unicellular eukaryote (*Acanthamoeba castellanii*, supergroup Amoebozoa)

**DOI:** 10.1016/j.dib.2014.08.005

**Published:** 2014-08-22

**Authors:** Ryan M.R. Gawryluk, Kenneth A. Chisholm, Devanand M. Pinto, Michael W. Gray

**Affiliations:** a Centre for Comparative Genomics and Evolutionary Bioinformatics, Department of Biochemistry and Molecular Biology, Dalhousie University, Halifax, Nova Scotia, Canada; b Mass Spectrometry and Proteomics Group, National Research Council of Canada, Halifax, Nova Scotia, Canada

## Abstract

This article describes and directly links to 1033 *Acanthamoeba castellanii* mitochondrial protein sequences. Of these, 709 are supported by Mass Spectrometry (MS) data (676 nucleus-encoded and 33 mitochondrion-encoded). Two of these entries are previously unannotated mtDNA-encoded proteins, which we identify as highly divergent mitochondrial ribosomal proteins. Our analysis corrects many *A. castellanii* protein sequences that were incorrectly inferred previously from genomic data deposited in NCBI.

**Table:** 

**Specifications table**	
Subject area	Biology

More specific subject area	Mitochondrial proteomics
Type of data	Tables (Excel spreadsheets), text files (FASTA sequence files)
How data were acquired	Tandem mass spectrometry (AB/MDS-SCIEX QTrap 4000 mass spectrometer)
Data format	Raw and analyzed
Experimental factors	Mitochondria from *Acanthamoeba castellanii* were prepared by subcellular fractionation. A portion was disrupted by passage through a French press. Soluble and insoluble (membrane) fractions were then separated by centrifugation. Both intact mitochondria and fractions enriched in soluble and membrane proteins were analyzed
Experimental features	Combined experimental (SCX-HPLC followed by MS/MS, or SDS-PAGE followed by MS/MS) and bioinformatics (data mining) approaches to identify mitochondrial proteins
Data source location	Halifax, Nova Scotia, Canada
Data accessibility	*Data are with this article and in the Data in Brief DataVerse repository* (http://thedata.harvard.edu/dvn/dv/dib) under study IDs: 26973, http://dx.doi.org/10.7910/DVN/26973[1] and 26980, http://dx.doi.org/10.7910/DVN/26980[2]

**Value of the data**

• We report 1033 *A. castellanii* mitochondrial protein sequences, 709 supported by MS/MS data (676 nucleus-encoded and 33 mitochondrion-encoded), including two previously unannotated mtDNA-encoded components, which we identify as highly divergent mitochondrial ribosomal proteins.
• All reported sequences are complete and have been verified against transcriptomic data and interpreted in [3].
• Our analysis corrects many *A. castellanii* protein sequences that were previously incorrectly inferred from genomic data deposited in NCBI.
• Mitochondrial targeting sequences (MTS) are inferred from MitoProt and/or TargetP prediction programs.

## 1 Data, experimental design, materials and methods

In-gel protein digestion, in-solution protein digestion, SCX-HPLC of peptides and MS/MS were performed as described in [4]. Precursor ions with charges of 2^+^ and 3^+^ were examined. Peptide sequences were assigned using Mascot (Matrix Science) to search an EST database using the following search parameters: MS and MS/MS mass tolerances were set to ±1.2 and ±0.6 Da, respectively. One missed cleavage was allowed and carbamidomethylcysteine and oxidized methionine were set as fixed and variable modifications, respectively. Searches were based on a significance threshold of *p*<0.05. MudPIT scoring was used to remove protein hits that had scores based purely on a large number of low-scoring peptide matches. Ion score cut-off was set at ≥25 and each protein hit was required to have at least one bold red (best match in database) peptide. False positive rates were calculated using the decoy option provided by Mascot and estimated as below 2%. Peptide sequences and inferred protein identities are compiled in Supplemental Table S1.

Annotation of proteins identified by MS/MS analysis was performed by querying a variety of databases (genomic and transcriptomic) using BLAST (BLASTp, tBLASTn and PSI-BLAST) algorithms [5]. Results are compiled in Supplemental Table S2 and Fasta files containing protein sequences [1]. These files are: (1) MS-identified mtDNA-encoded proteins; (2) MS-identified nuDNA-encoded mitochondrial proteins; (3) MS-identified putative nuDNA-encoded non-mitochondrial proteins; (4) putative mitochondrial proteins identified by bioinformatic means (*in silico*) only.

Some of these data have previously been published [6–9].

A Mascot semi-tryptic peptide search was employed to detect putative mature protein N-termini, essentially as described [10]. Briefly, semi-tryptic peptides with ion scores surpassing the Mascot identity threshold and lacking an N-terminal Arg or Lys tryptic cleavage site were considered. If a semi-tryptic peptide lacking an N-terminal Arg or Lys was located in the N-terminal region of the inferred protein sequence (usually the first 50–60 amino acids) and not located in a region of the protein conserved in other species, it was considered to be the putative mature protein N-terminus [2].

OrthoMCL [11] was used to identify orthologs/co-orthologs of *A. castellanii* proteins in the predicted cellular proteomes of *Arabidopsis thaliana* (35,386 sequences from TAIR 10 protein dataset), *Homo sapiens* (20,270 sequences from UniProt), *Saccharomyces cerevisiae* (6,572 sequences from *Saccharomyces* Genome Database), and *Tetrahymena thermophila* (27,054 sequences from NCBI nr database), all of which have been examined intensively at the level of the mitochondrial proteome. For *A. castellanii* protein sequences, we used a six-frame translation of RNA-seq data supplemented with corrected versions of each mitochondrial protein identified in our analysis. OrthoMCL was run according to the recommended parameters, with an *E*-value threshold of 1e−5. Data are compiled in Supplemental Table S3.

## Supplementary materials

Supplementary data Supplemental Table S1: A list of peptide sequences and associated statistics for mtDNA-encoded (A) and nuDNA-encoded (B) mitochondrial proteins, as well as putative contaminant proteins (C), identified by tandem mass spectrometry (MS/MS).

Supplementary data Supplemental Table S2: Complete nuDNA- (“N”) and mtDNA- (“M”) encoded protein sequences identified by MS/MS, along with putative mitochondrial protein sequences identified bioinformatically (“B”)..

Supplementary data Supplemental Table S3: A summary of the results of an OrthoMCL analysis to identify orthologs of *A. castellanii* mitochondrial proteins (identified by both MS/MS and purely bioinformatic approaches) in a land plant (*Arabidopsis thaliana*), animal (*Homo sapiens*), fungus (*Saccharomyces cerevisiae)* and ciliate protozoon (*Tetrahymena thermophila*).

## Footnotes

Appendix A Supplementary data associated with this article can be found in the online version at doi:10.1016/j.dib.2014.08.005.
